# Metal artifact reduction on musculoskeletal CT: a phantom and clinical study

**DOI:** 10.1186/s41747-023-00354-9

**Published:** 2023-07-31

**Authors:** Petter Midthun, Eva Kirkhus, Bjørn Helge Østerås, Per Reidar Høiness, Andrew England, Safora Johansen

**Affiliations:** 1 Health Faculty, Oslo Metropolitan University, Pilestredet 48, 0130 Oslo, Norway; 2 Department of Radiology and Nuclear Medicine, Oslo University Hospital, Oslo, Norway; 3 Department of Physics and Image Analysis, Oslo University Hospital, Oslo, Norway; 4 Department of Orthopaedics, Drammen Hospital, Drammen, Norway; 5 School of Medicine, University College Cork, Cork, England; 6 Department of Cancer Treatment, Oslo University Hospital, Oslo, Norway

**Keywords:** Algorithms, Artifacts, Image processing (computer-assisted), Phantoms (Imaging), Tomography (x-ray computed)

## Abstract

**Background:**

Artifacts caused by metal implants are challenging when undertaking computed tomography (CT). Dedicated algorithms have shown promising results although with limitations. Tin filtration (Sn) in combination with high tube voltage also shows promise but with limitations. There is a need to examine these limitations in more detail. The purpose of this study was to investigate the impact of different metal artefact reduction (MAR) algorithms, tin filtration, and ultra-high-resolution (UHR) scanning, alone or in different combinations in both phantom and clinical settings.

**Methods:**

An ethically approved clinical and phantom study was conducted. A modified Catphan® phantom with titanium and stainless-steel inserts was scanned with six different MAR protocols with tube voltage ranging from 80 to 150 kVp. Other scan parameters were kept identical. The differences (∆) in mean HU and standard deviation (SD) in images, with and without metal, were measured and compared. In the clinical study, three independent readers performed visual image quality assessments on eight different protocols using retrospectively acquired images.

**Results:**

Iterative MAR had the lowest ∆HU and ∆SD in the phantom study. For images of the forearm, the soft tissue noise for Sn-based 150-kVp UHR protocol with was significantly higher (*p* = 0.037) than for single-energy MAR protocols. All Sn-based 150-kVp protocols were rated significantly higher (*p* < 0.046 than the single-energy MAR protocols in the visual assessment.

**Conclusions:**

All Sn-based 150-kVp UHR protocols showed similar objective MAR in the phantom study, and higher objective MAR and significantly improved visual image quality than single-energy MAR.

**Relevance statement:**

Images with less metal artifacts and higher visual image quality may be more clinically optimal in CT examination of musculoskeletal patients with metal implants.

**Key points:**

• Metal artifact reduction algorithms and Sn filter combined with high kVp reduce artifacts.

• Metal artifact reduction algorithms introduce new artifacts in certain metals.

• Sn-based protocols alone may be considered as low metal artifact protocols.

**Graphical Abstract:**

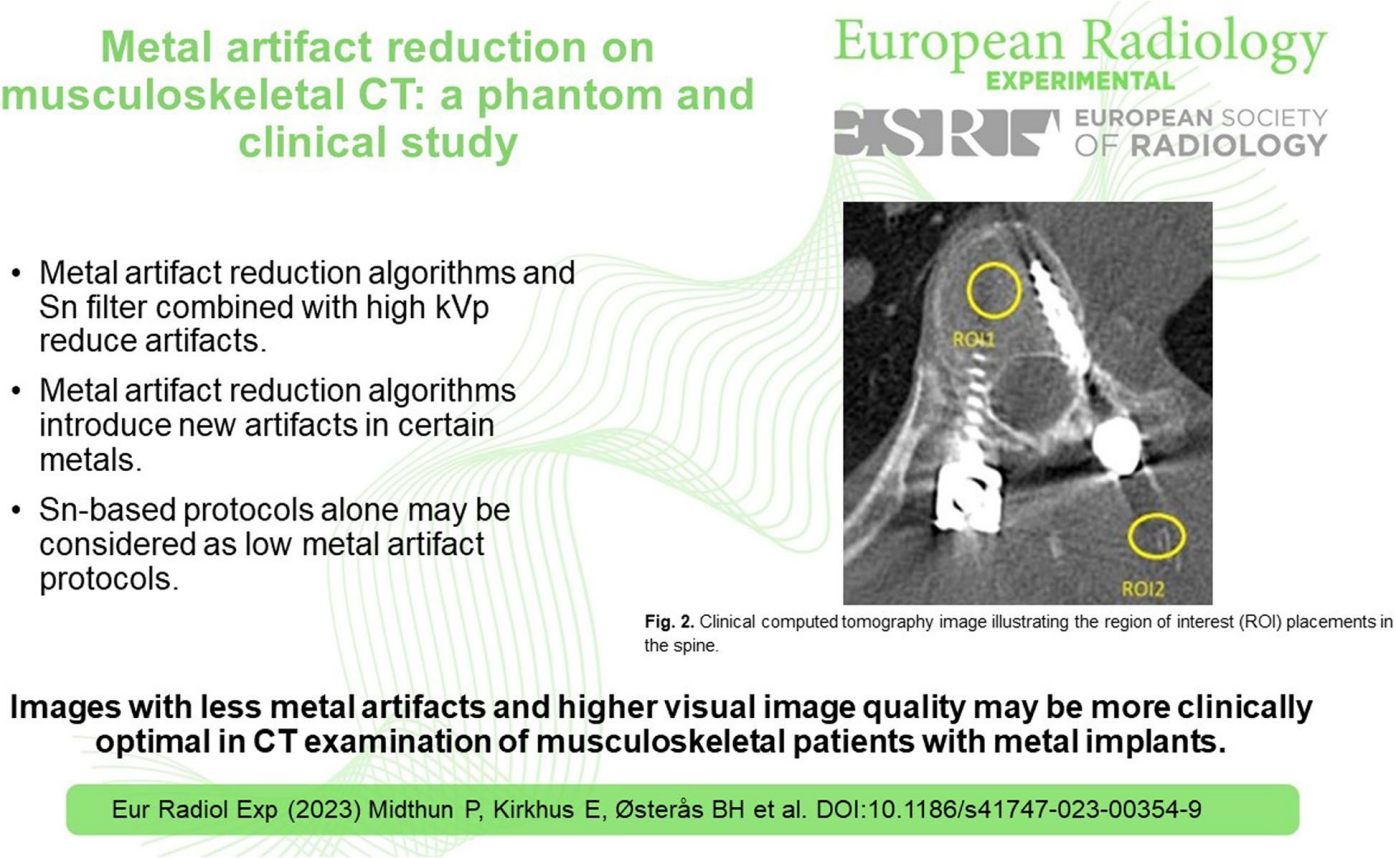

## Background

Artifacts caused by metal, such as orthopedic implants, surgical clips, coils, wires, and dental fillings on computed tomography (CT) can lead to interpretation difficulties [1]. Relevant anatomical structures are often obscured by such artifacts, which can increase the risk of missing findings [2]. The intensity of these artifacts depends on the composition, size, and implant location along with scanner acquisition and reconstruction parameters [3, 4]. Beam hardening, x-ray scatter, edge effects, noise, photon starvation and the combination of these effects are the primary causes of artifacts, which presents as dark and bright streaks and/or shading artifacts [1–4]. There are some techniques, which can reduce metal artifacts such as increasing the tube voltage and filament current resulting in more photons reaching the detector and subsequently reducing beam hardening and photon starvation artifacts [1]. However, without altering other scan parameters these approaches increase the radiation dose to the patient and provide only limited improvement of image quality [2]. Softer reconstruction kernels or iterative reconstruction algorithms reducing image noise and potentially reduce the impact of metal artifacts, but these approaches reduce spatial resolution [1, 5, 6].

Several metal artifact reduction (MAR) software methods have been introduced and are available on contemporary CT scanners [7]. Projection-based algorithms act in projection space and replace projections with low signal caused by metal, with interpolation from neighboring projections [8]. These algorithms primarily suppress artifacts that are caused by photon starvation [2]. There are many software packages available, such as SmartMAR (General Electric Healthcare), O-MAR (Philips Healthcare), iMAR (Siemens Healthineers), and SEMAR (Canon Medical Systems) [7]. These algorithms detect and segment the metal from the original image by using a Hounsfield unit (HU) threshold and compensates the image with a designated algorithm for photon starvation caused by the metal implants resulting in reduced artifacts [7–9]. Altering the visual representation of metal and artifacts introduced by the algorithms has been reported [7–9]. The two MAR software algorithms in our study, iMAR and SEMAR limits the ability to reconstruct images with the sharpest kernels which results in lower spatial resolution. For Siemens iMAR and Canon SEMAR, selecting MAR reconstruction limits the sharpness of kernels available Br59 (Siemens) and FC30 (Canon) [8].

Another method to reduce artefacts related to metal implants is the introduced additional built-in tin filter [10]. The introduction of tin filters, in recent years, has been used to support ultra-low dose CT scanning [10]. In this situation the tin filter increases the beam energy by removing low energy photons, making the beam less susceptible to beam-hardening [10]. This can be applied in conjunction with high tube potential (140 or 150 kVp) and iMAR algorithms (Siemens Healthineers, Erlangen, Germany). The higher mean photon energy results in higher probability of x-ray photons available to the detector [10].

Furthermore, an ultra-high resolution (UHR) CT scan mode has also been released [11]. UHR scanning is achieved using an attenuating filter, which can be arranged to partially cover the detector surface in advance of scanning [11]. This ‘comb’ filter improves the spatial resolution by reducing each detector cell’s aperture width at the expense of dose efficiency [11]. Combined with tin filtration and a high tube voltage this scan method could result in low artifact images but combined with high spatial resolution as it is not limited by software [10, 11]. Due to UHRs kernel selection (Ur) it is not possible to combine UHR scans with iMAR reconstruction at any kernel level [11]. The impact of UHR scanning when combined with tin filtration and high tube potential on metal artifact reduction scans has not yet been investigated.

The aim of this study is to investigate the MAR capability of SEMAR, iMAR, tin filtration (Sn) and UHR alone or in different combinations, across a range of tube potentials when scanning a phantom. Additionally, we retrospectively compared the objective and visual image quality of CT images in patients with metal implants scanned with five Sn-based and three SEMAR clinical protocols to recommend an optimum MAR protocol.

## Methods

### Phantom study

The investigation of the MAR capability of SEMAR on a 640-slice Canon Aquilion Genesis (Canon Medical Systems, Otawara, Tochigi, Japan) and tin filtration, iMAR and UHR on a 384-slice Siemens Somatom Force (Siemens Healthineers, Erlangen, Germany) was performed in this study. A modified Catphan® phantom (The Phantom Laboratory, Salem NY, USA) was used. The Catphan® 710 phantom contains two metal inserts (13-mm diameter): one of titanium and one of stainless steel. Only one metal could be inserted at a time. The inserts used for measurements were bone and soft tissue equivalent.

Detailed descriptions of scan parameters used in both phantom and clinical study are shown in Table 1. The different tube potentials for Sn scans and Canon scans were due to scanner limitations. iMAR reconstruction were set to extremity implants setting. No reconstruction preset was available for SEMAR. For Siemens the modular transfer function values at 50% were 8.3 and 16.6 (line pairs/cm) for Br59 kernel and Ur77 kernel, respectively. The modular transfer function value for the FC 30 kernel on the Canon scanner was 9.7 (line pairs/cm) at 50%. The Catphan® phantom was scanned three times per technique combination: non-metal scans were considered reference scans and then the scans were repeated with titanium and stainless-steel inserts. The mAs values of all scans were modified to provide a CT dose index volume (CTDI_vol_) of 4.5 mGy.

**Table 1:** CT scan protocols used for phantom study and the retrospective case analysis

Protocol	Scanner manufacturer	Body part	kVp	mAs	Slice thickness (mm)	Pitch	Rotation time (s)	Collimation	Iterative	Kernel	Sn	MAR
Phantom study												
UHR	Siemens		80, 100, 120, 140, 150	281,127, 77, 52, 45	1.0	1.0	1.0	64 × 0.6	Admire 1	UR 77	No	None
iMAR	Siemens		80, 100, 120, 140, 150	232,107, 66, 43, 37	1.0	1.0	1.0	192 × 0.6	Admire 1	Br 59	No	iMAR
Sn	Siemens		100, 150	1065, 151	1.0	1.0	1.0	192 × 0.6	Admire 1	Br 59	Yes	None
Sn plus UHR	Siemens		100, 150	885, 169	1.0	1.0	1.0	64 × 0.6	Admire 1	Ur 77	Yes	None
Sn plus iMAR	Siemens		100, 150	1065, 151	1.0	1.0	1.0	192 × 0.6	Admire 1	Br 59	Yes	iMAR
SEMAR	Canon		80, 100, 120, 135	580, 270, 150, 110	1.0	1.0	1.0	0.5SR × 0.25	AIDR 3D STD	FC 30	No	SEMAR
Clinical study												
1	Siemens	Forearm	150	220^a^	1.0	0.55	1.00	64 × 0.6	ADMIRE 1	Ur77^d^	Yes	None
2 and 2*	Siemens	Spine	150	400^b^	1.0	0.80	0.50	192 × 0.6	ADMIRE 3	Br59^e^	Yes	IMAR
3 and 3*	Siemens	Sacroiliac joint	150	145^b^	1.0	0.80	1.00	192 × 0.6	ADMIRE 2	Br59^e^	Yes	IMAR
4	Canon	Forearm	135	150^a^	1.0	0.80	0.50	0.5SR × 0.25	AIDR 3D STD	FC30^f^	No	SEMAR
5	Canon	Spine	135	80/580^b,c^	1.0	0.80	0.50	0.5SR × 0.25	AIDR 3D STD	FC30^f^	No	SEMAR
6	Canon	Sacroiliac joint	135	50/850^b,c^	1.0	0.80	0.75	0.5SR × 0.25	AIDR 3D STD	FC30^f^	No	SEMAR

2* and 3* protocols: Sn only and Sn combined with iMAR images are derived from the same scan, therefore protocols 2 and 3 are Sn only protocols, while protocols 2* and 3* are Sn plus iMAR protocols

*ADMIRE* Advanced modeled iterative reconstruction, *AIDR 3D STD* Adaptive iterative dose reduction 3D standard, *iMAR* Iterative metal artifact reduction, *MAR* Metal artifact reduction, *SEMAR* Single-energy metal artifact reduction, *Sn* Tin filter

^a^Effective mAs selected for scans without dose modulation

^b^Selected reference mAs for scans using automatic dose modulation

^c^Minimum and maximum mAs values for Canon scanner

^d^Ur77

^e^Br59 are the sharpest possible kernel selection for Siemens images

^f^FC30 is the sharpest possible kernel selection for Canon images

### Objective image quality assessment (phantom study)

**Fig. 1:**
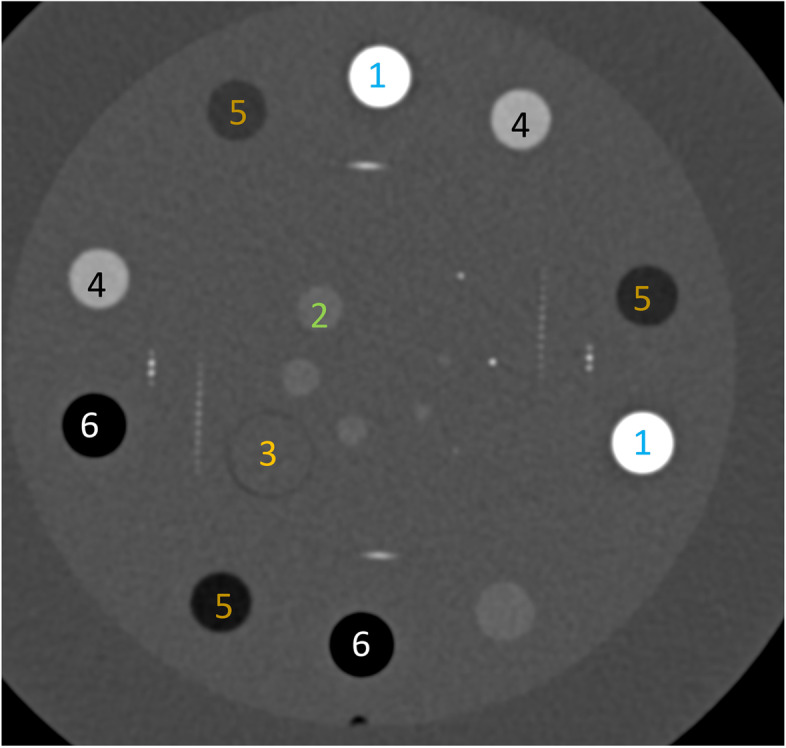
Region of interest (ROI) placement and measurement in the included inserts in the Catphan® phantom inserts: ROI1 in bone (1), and ROI2 in soft tissue (2). 1 Bone insert. 2 Soft tissue insert. 3 Area for metal insert. 4 Iodine insert. 5 Liquid insert. 6 Air insert

Phantom images were analyzed objectively using a Sectra Picture archiving and communication system (PACS) (Sectra AB, Linköping, Sweden). Regions of interest (ROIs) were placed in their respective inserts as shown in Fig. 1. ROI1 was measured in the bone insert and ROI2 in the soft tissue insert which was close to the metal. ROI1 was 9 mm in diameter while ROI2 was only 6 mm due to smaller size of soft tissue insert. Both ROIs were in the direct path of artifacts as the metal artifacts were evenly distributed throughout the image.

Mean HU and the standard deviation (SD) inferred as noise were measured on images, with and without metal in both the bone and soft tissue, for all phantom scans. Mean HU difference (ΔHU) and SD difference (ΔSD) were results of mean HU and SD in images with metal subtracted from mean HU and SD in images with no metal (baseline images).

### Clinical study

Patients with CT examinations of their orthopedic metal implants using different available protocols designed to minimize metal artifact in their first and in their second routine follow-up examinations, were identified on the PACS. This portion of the study was approved by the Data Protection Officer at the chosen Hospital (approval No. 21/14061). Our department had three available Sn filter-based protocols, one in combination with UHR for forearm, and two Sn only-based protocols for spine and sacroiliac joint with iMAR reconstruction marked with * after protocol number. SEMAR-based protocols for the same body parts were in use for the Canon scanner. Detailed descriptions of all scan protocols in this study are shown in Table 1. All patients would have undergone the examination with the same protocol regardless of this study. As this is a retrospective study, the authors had no impact on the different protocols and its scan parameters. The iMAR reconstruction presets were set to extremity implants for forearm and spine implants for spine and sacroiliac joint images. Again, CTDI_vol_ values were recorded.

### Objective image quality assessment (clinical study)

**Fig. 2:**
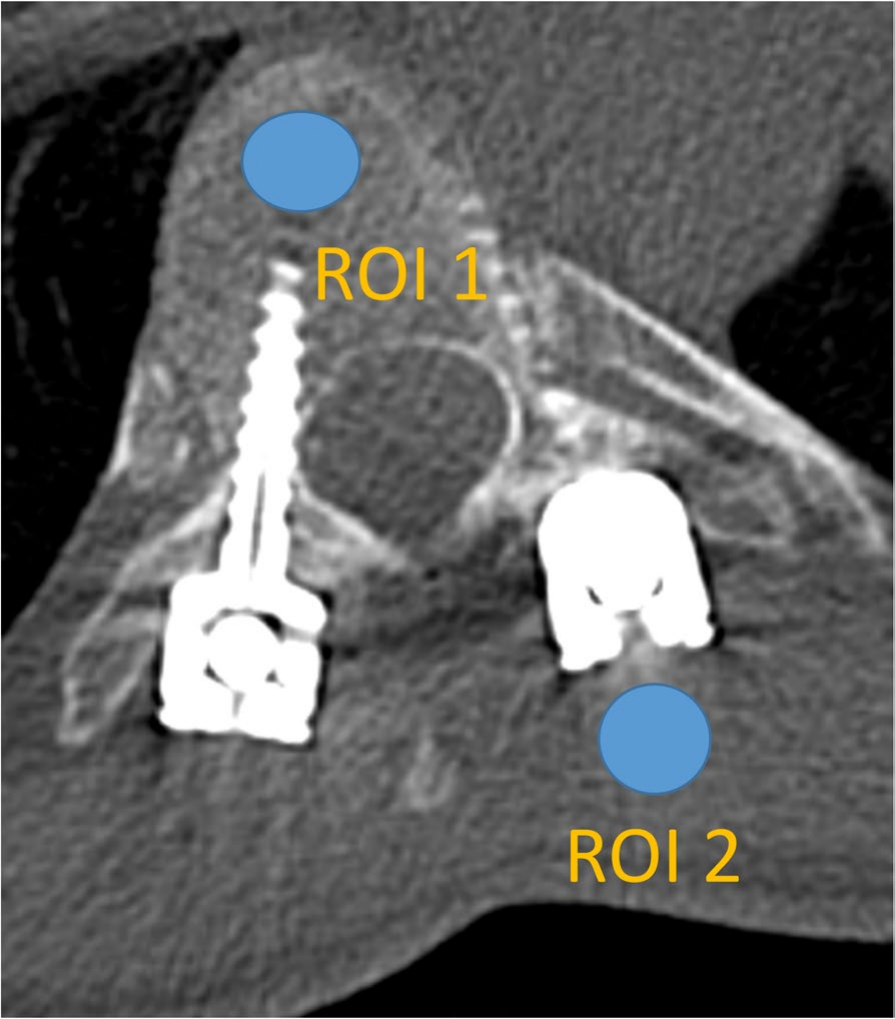
Clinical computed tomography image illustrating the region of interest (ROI) placements in the spine. The scan protocol included 150-kVp Sn-filter on a Siemens Somatom Force scanner. ROI1 is placed in the 7th thoracic vertebrae between metal implants and ROI2 in muscle

Objective image quality assessment of the clinical images was measured using two ROIs: ROI1 in bone, ROI2 in soft tissue or adjacent soft tissue (Fig. 2). ROI1 and ROI2 were placed as close to the metal as possible. To obtain comparable setup across body parts and different metals, the ROI placement was defined as ROI1 bone placed in the same bone as the metal was placed, and ROI2 soft tissue placed in muscle close to the metal. ROIs were 10 mm in diameter. Mean HU and SD values were measured and recorded. Median values of all HU and SD values were also recorded.

### Visual image quality assessment (clinical study)

For the visual image assessment (VIA) of the clinical images, all the images were anonymized, randomized, and presented to three observers; two radiologists (readers 1 and 2) and one orthopedic surgeon (reader 3), all with more than 15 years of clinical experience. The VIA consisted of the following criteria that radiologists use to evaluate typical patient cases [12]: (1) qualitative noise; (2) visualization of the metal implant; (3) visualization of periprosthetic cortical bone; (4) visualization of periprosthetic soft tissue; (5) extent of artifacts; and (6) diagnostic confidence. Likert scale scores from 0 to 5 with 5 being the highest possible score per each criteria indicating a perfect image in relation to the specific criteria, such as no noise (criterion 1) or no artifacts (criterion 5). Scale criteria 2, 3, and 4 focused on the visualization of metal itself and the tissue in close proximity [7, 12, 13]. Scoring was performed using Sectra PACS. Criteria 1, 5, and 6 focused on an overall impression of the image quality. Readers were provided with full image datasets reconstructed with the scanners respective bone algorithm and were allowed to adjust the window settings to individual preferences.

### Data analysis and statistics

Intra-reader and inter-reader agreements were assessed using quadratic weighted Fleiss κ for three or more readers and Cohen κ when comparing scores from two readers or different scores from the same reader [14]; κ scores of 0.00−0.20 were rated as slight agreement, 0.20−0.40 as fair, 0.41−0.60 as moderate, 0.61−0.80 as substantial, and 0.80−1.00 as almost perfect [14]. Median VIA scores were a result of all scores given for each criterion. No weighing or total scores were used for the VIA. Two-sided statistical tests were performed to compare differences in image quality differences in the clinical image quality assessment; *p* values < 0.05 were considered as statistically significant. For statistical analysis, Stata (v16.1) (StataCorp LLC, College Station, TX, USA) was used. The descriptive analysis performed in this study includes among other the median and mean HU and SD.

## Results

### Objective image assessment (phantom study)

**Fig. 3:**
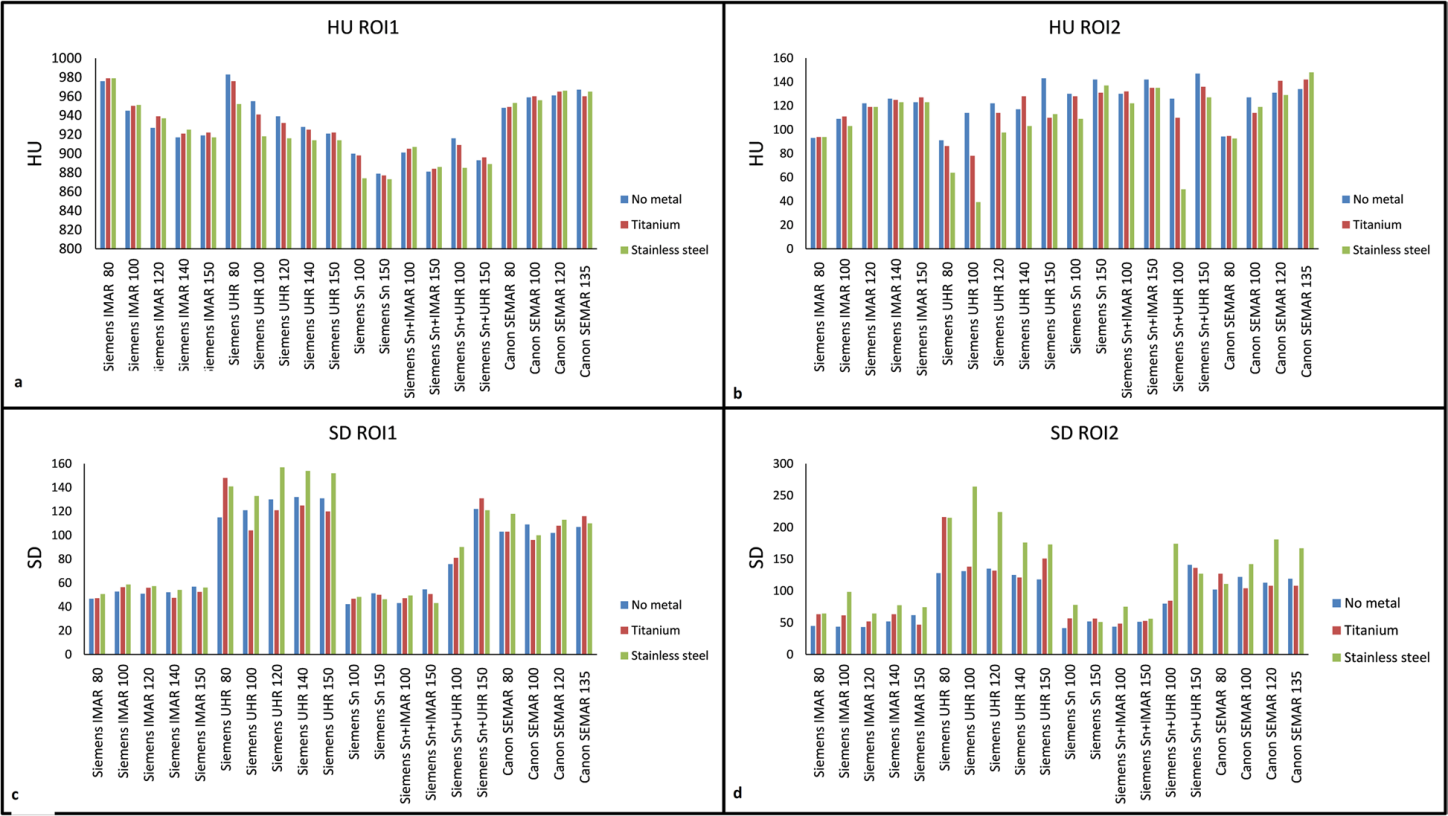
**a** Mean HU observed in the bone insert (ROI1). **b** Mean HU observed in the soft tissue insert (ROI2). **c** Noise (SD) observed in the bone insert (ROI1). **d** Noise (SD) observed in the soft tissue insert (ROI2). *ROI* Region of interest, *SD* Standard deviation

**Fig. 4:**
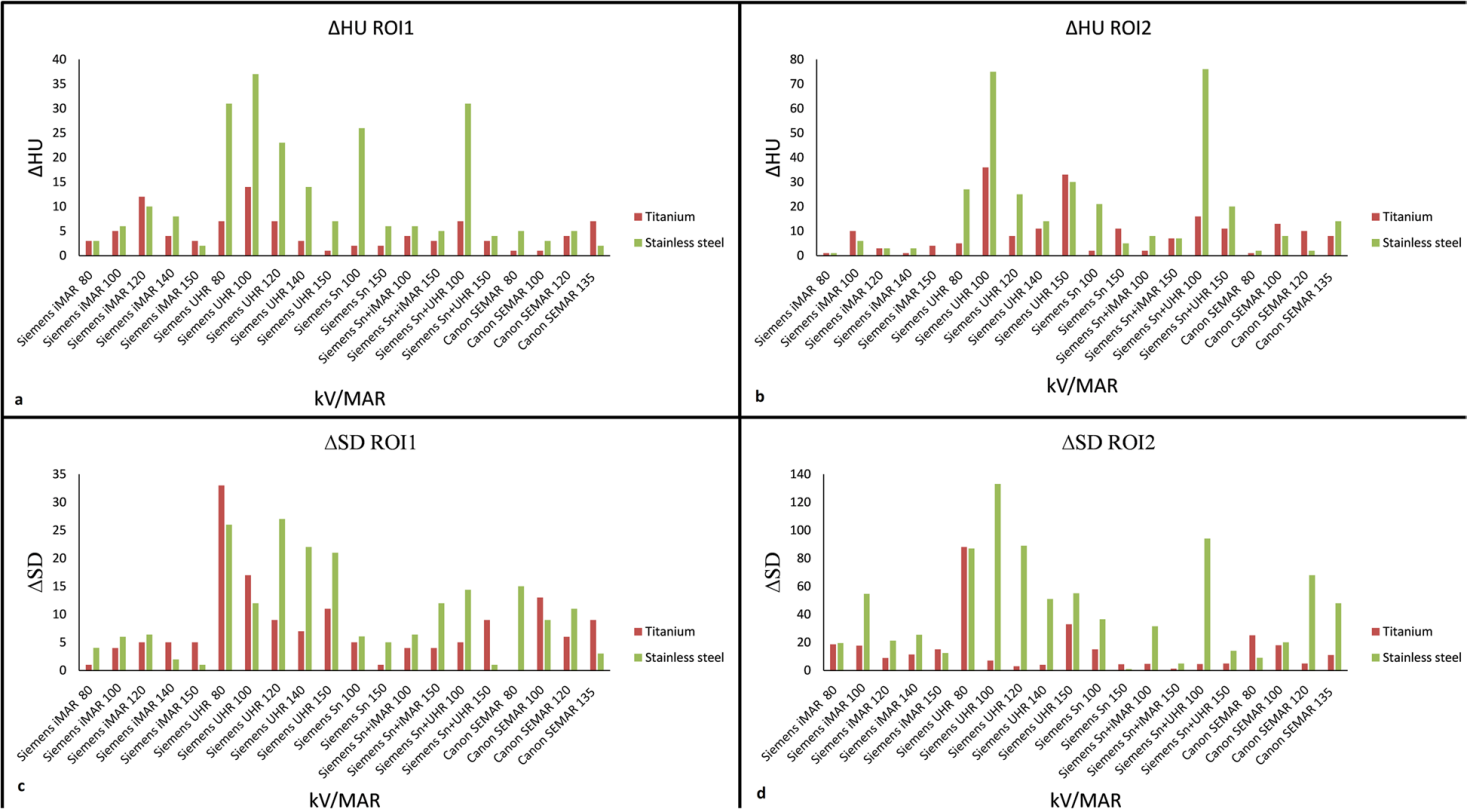
**a** ∆HU in the bone insert (ROI1). **b** ∆HU in the soft tissue insert (ROI2). **c** ∆noise (SD) in bone insert (ROI1). **d** ∆noise (SD) in soft tissue insert (ROI2). *ROI* Region of interest, *SD* Standard deviation

An identical CTDI_vol_ of 4.5 mGy for all protocols and scans in the phantom study was recorded. The mean HU and SD for all the included protocols in the phantom study are shown in Fig. 3a–d. The lowest ∆HU and ∆SD in phantom-based scans when comparing scans with and without metal were observed with Siemens iMAR 150 kVp (Fig. 4) in both the bone and soft tissue inserts for both type of metals. However, the ∆HU differences between Siemens iMAR 150 kVp, Siemens iMAR 80 or 140 kVp, and Canon SEMAR 80 kVp were all lower than 5 HU. A ∆SD lower than 5 HU was also observed when comparing Siemens Sn 150 kVp and Siemens Sn plus iMAR 150 kVp. For other protocols included in this study, the ∆HU and ∆SD were slightly higher with stainless metal compared to titanium (Fig. 4).

### Objective image assessment (clinical study)

In the 30 patients imaged from 2018 to 2022, there were seven different metal compositions based on titanium and stainless steel. Implants also ranged in thickness and were implanted in three body areas. The first CT examination was usually performed the day after the implant was inserted while the second CT scan was usually performed 1−2 years later as part of routine follow-up. Patients experiencing abnormality in form of pain and/or suspected implant loosening were examined earlier. A total of 60 CT examinations in 30 patients were retrospectively included in the clinical part of this study. Full details of the patients, metal characteristics, and radiation dose are provided in Table 2.

**Table 2:** Patient and metal implant characteristics for the clinical study

**Variable**	**Number (%), mean/median and ± standard deviation/range**	
Number of participants	30 (100%)	
Male	11 (42%)	
Female	19 (58%)	
Age (years)	45.5/50.0 ± 15.6 (range 17.0−70.0)	
Time between examinations (months)	10.4/10.5 ± 5.5 (range 3.0−23.0)	
*Metal type*		
Titanium	23 (77%)	
Stainless steel + titanium	7 (23%)	
*Body parts and metal type*		
7 forearm (23%)	4 spine (13%)	19 sacroiliac joint (63%)
Metal type and thickness	Metal type and thickness,	Metal type and thickness
3 stainless steel LCP plates 3.5 mm 2 stainless steel LCP plates 2.5 mm 2 stainless steel LCP plates 3.5 mm 6 − 30 titanium screws	Stainless steel rods 5 mm 4−16 titanium screws	4 × 3 Rialto titanium screws 12 mm 9 × 3 Ifuse titanium screws 12 mm 6 × 6 Ifuse titanium screws 12 mm
*Clinical indications for implants*		
Fracture/accident	7 (24%)	
Pelvic pain/instability	18 (60%)	
Other	5 (16%)	
*Indication for second CT examination*		
Routine follow-up	20 (67%)	
Pain in relation to implant	10 (33%)	
*Dose (mGy)*		
*CTDI*_***vol***_ ***(mGy)***	Siemens mean/median, ± SD and range	Canon mean/median, ± SD and range
*Forearm*	5.1/5.1 ± 0.4 (range 4.7−5.8)	7.1/7.5 ± 1.9 (range 4.4−40.4)
Spine	7.7/6.9 ± 1.7 (range 6.2−10.7)	5.6/5.1 ± 1.4 (range 4.4−8.0)
Sacroiliac joint	6.4/5.1 ± 2.9 (range 3.4−13.9)	6.8/5.1 ± 2.8 (range 4.8−12.8)
*Protocols*	Protocol number Siemens	Protocol number Canon
Forearm	1	4
Spine	2 and 2*	5
Sacroiliac joint	3 and 3*	6

2* and 3* refer to Siemens Sn plus iMAR scan protocols

*CT* Computed tomography, *CTDI*_*vol*_ CT dose index volume, *LCP* Locking compression plate

**Fig. 5:**
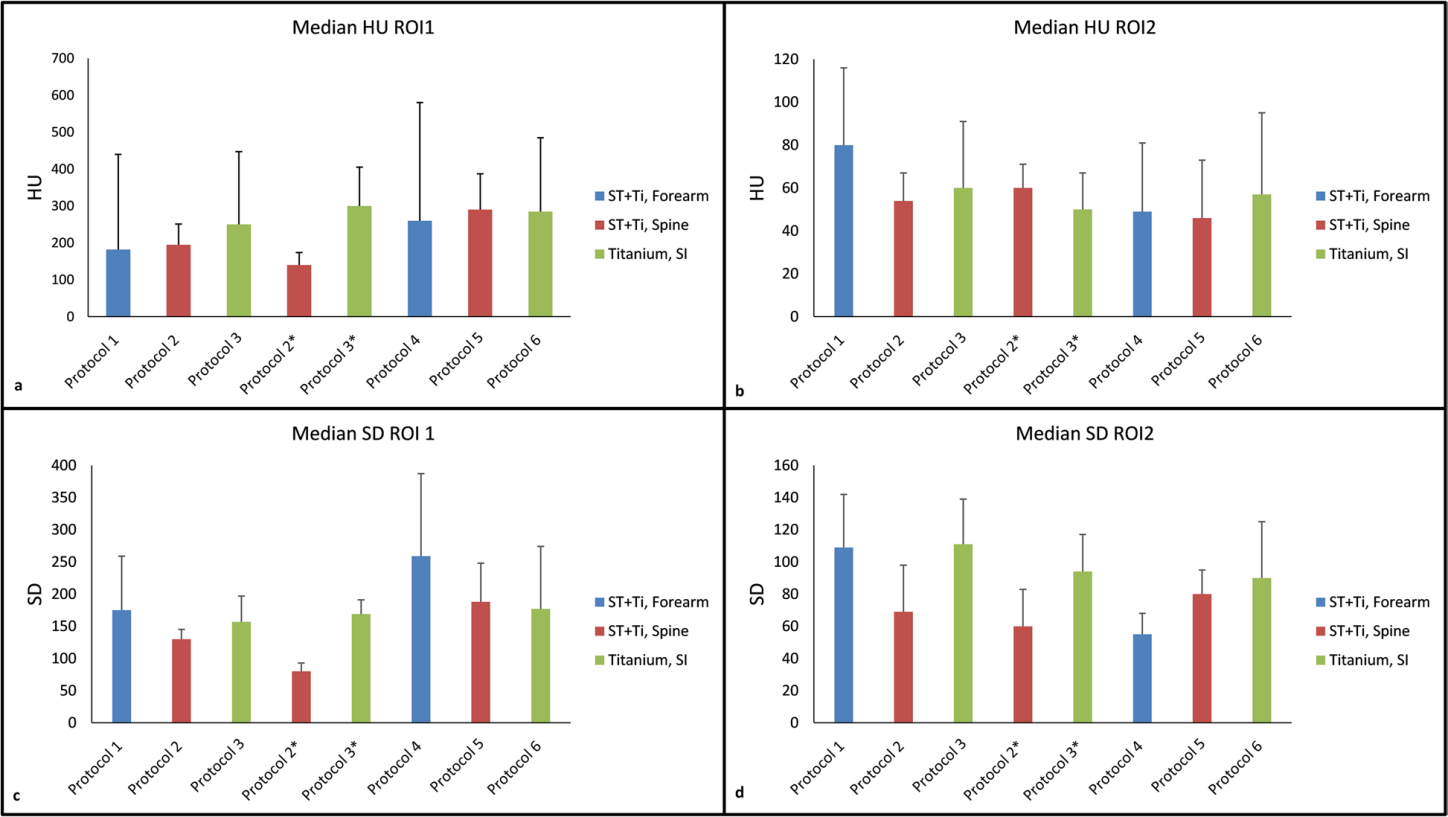
**a** Observed median HU in bone (ROI1). **b** Observed median HU in low soft tissue (ROI2). **c** Observed median noise (SD) in bone (ROI1). **d** Observed median noise (SD) in soft tissue (ROI2). The error bars indicate standard deviation. *ROI* Region of interest, *SD* Standard deviation

The highest median HU of 637 and 97 were measured in bone and soft tissue, respectively, for patients scanned with protocol 3 and protocol 6 with three titanium “Rialto” screws, which are used to fuse the sacroiliac joint together in order to reduce pain in patients with lower back pain [15]. The lowest median HU of 140 and 46 were observed in patients scanned with protocol 2 and protocol 5 in bone and soft tissue, respectively. However, these differences were not statistically significant in any case (*p* ≥ 0.2202). Median scores for all protocols are shown in Fig. 5.

The measured median SD was highest (109) in images scanned using protocol 1 with a significantly difference (*p* = 0.037) when compared to SD (55) in images obtained using protocol 4 in soft tissue (Fig. 5d).

The highest median SD (373) was observed in patients with six titanium “Ifuse” screws which has the same purpose and function as Rialto but made from another manufacturer [16] scanned with protocol 6 when compared to the lowest SD (113) measured in protocol 2 in the spine with stainless steel rods and titanium screws in bone. For soft tissues, protocol 6 resulted in the highest and lowest median SD of 130 and 47, in the six titanium Ifuse screws and three titanium Rialto screws, respectively.

Metal composition had no significant impact on CTDI_vol_ levels for any protocol or body part (all *p* ≥ 0.489). There were no significant differences in mean CTDI_vol_ values between vendors (*p* ≥ 0.319), as seen in Table 2.

### Visual image assessment (clinical study)

The intra-reader agreement was moderate for one reader (reader 3) and substantial for the two other readers (readers 1 and 2) with κ values of 0.58, 0.68, and 0.71, respectively. The inter-reader agreement for VIA showed a κ value of 0.24. In the VIA all Siemens Sn 150-kVp, protocols 1, 2, and 3 and protocols 2* and 3* were rated significantly higher ***(*p* ≤ 0.04615) than all Canon SEMAR 135-kVp protocols 4, 5, and 6 (Table 3).

**Table 3:** Median visual image assessment scores for each criterion across all readers

**Protocol number (see **Table 1**)**	**Qualitative noise (criterion 1)**	**Visualization of metal implant (criterion 2)**	**Visualization of periprosthetic cortical bone (criterion 3)**	**Visualization of periprosthetic soft tissue (criteri**on** 4)**	**Extent of artifacts (criterion 5)**	**Diagnostic confidence (criterion 6)**
Median score for all readers						
** 1**	3	3	3	3	2	3
** 2**	4	4	4	3	4	4
** 3**	3	3	4	3	3	4
2*	4	3	3.5	3.5	4	4
3*	3	3	3	3	3	4
** 4**	3.5	3	2.5	2.5	2	3
** 5**	3	2	2	2	2	2.5
** 6**	2	2	3	3	2	3

2* and 3* refer to Siemens Sn plus iMAR scan protocols

**Fig. 6:**
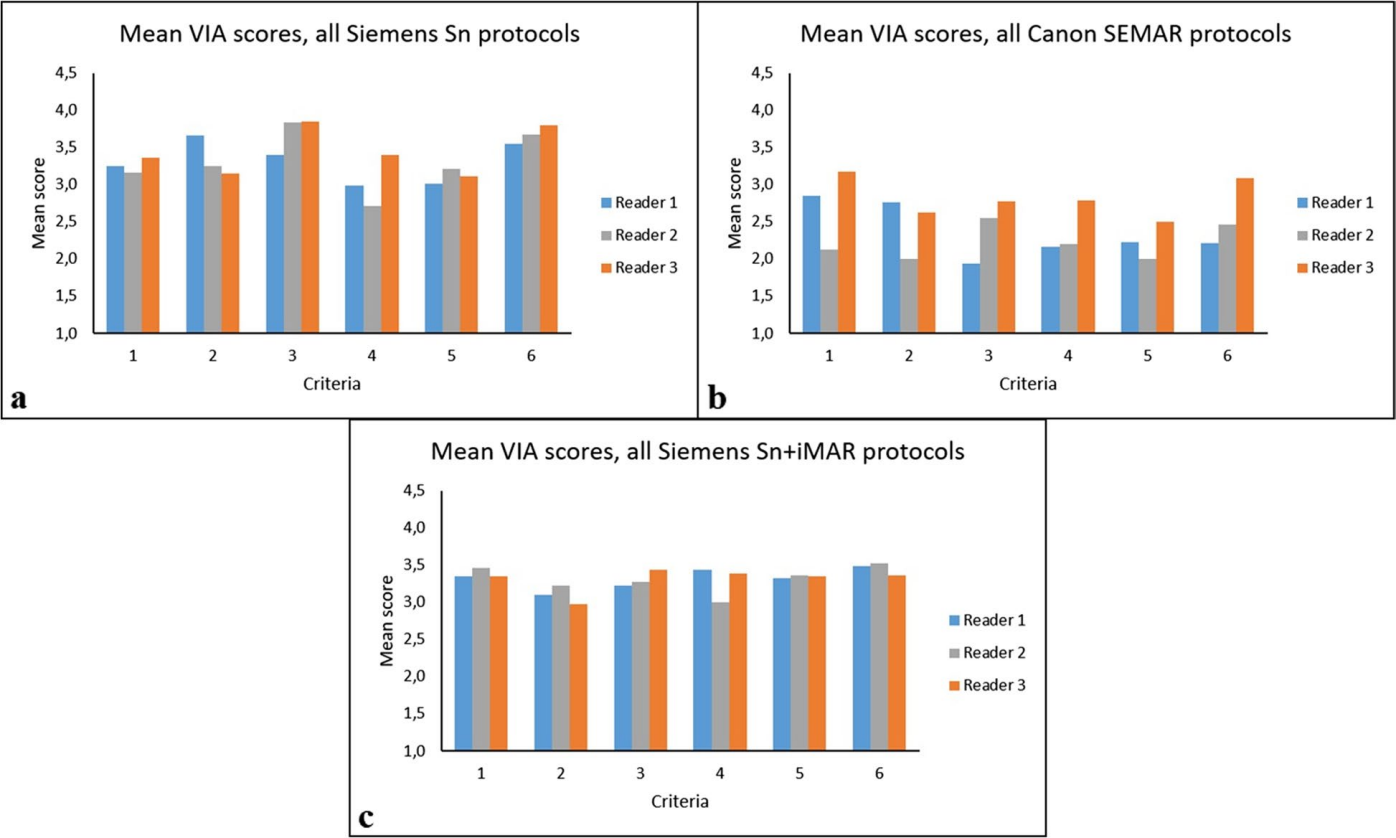
**a** Separate mean visual image assessment (VIA) scores for each of the three readers in all Siemens Sn-based protocols. **b** Mean VIA scores in all Canon SEMAR protocols. **c** Mean VIA scores in all Siemens Sn plus iMAR protocols

For the spine and sacroiliac joint, using protocols 2 and 3 (Table 3) received the highest median VIA scores, from all three readers, for all criteria. This difference was statistically significant (*p* ≤ 0.005) compared to other included protocols (Table 3). Median VIA scores for all three readers and the included protocols are shown in Fig. 6.

**Fig. 7:**
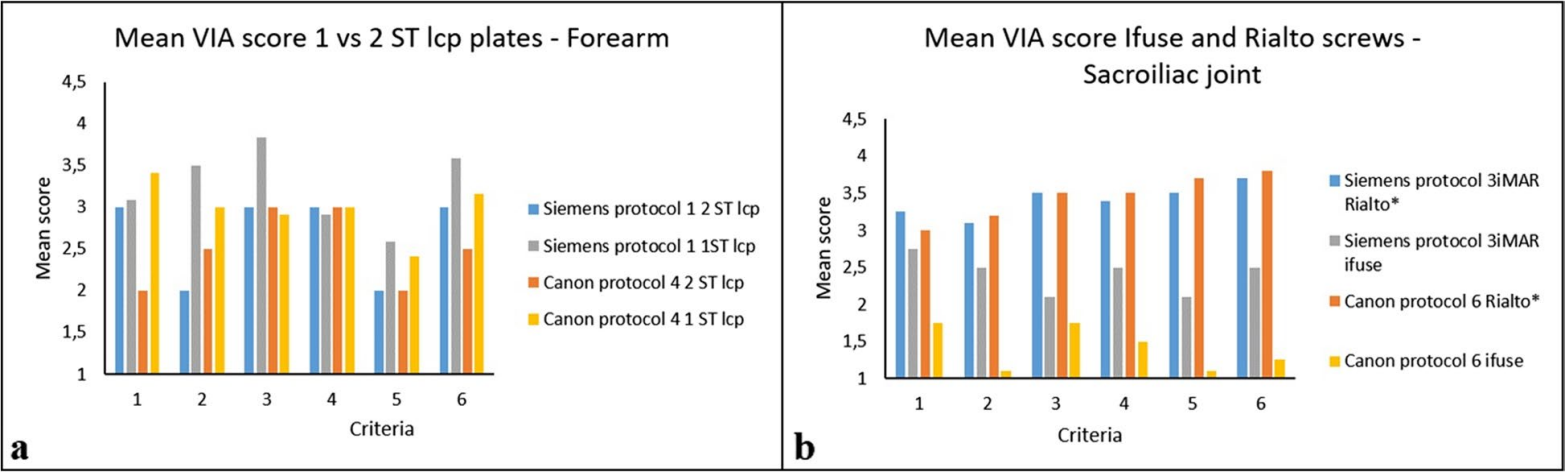
**a** Mean visual image assessment (VIA) scores for all readers rating forearm with two stainless steel plates *versus* one for protocol 1 and Canon protocol 4. **b** Mean VIA scores for protocol 6 and protocol 3* for all 3 readers rating sacroiliac joints with titanium Rialto or Ifuse screws. *Indicates statistical significant improvement of VIA scores in images containing rialto screws *versus* Ifuse (*p* < 0.005)

In respect to implant types and thickness, the median VIA scores were similar when using the Siemens protocol 1 *versus* Canon protocol 4 in the forearm (*p* = 0.023). Furthermore, the three Rialto screws in the sacroiliac joint received higher median VIA scores compared to three and six Ifuse screws in the same body part (*p* = 0.0015) using protocol 3 and protocol 6 (Fig. 7b).

## Discussion

In this study, Siemens iMAR and Canon SEMAR algorithms showed slightly increased MAR on objective assessment compared to other scan protocols. However, the non-MAR software Siemens 150-kVp Sn resulted in highest median VIA score in the visual assessment, for all body parts and metal compositions. More accurate HU and SD results do not necessarily lead to higher visual assessment scores, implying more factors impacted the image quality than those covered in the objective part of this study. Unfortunately, there is currently no standardized way to describe and grade the extension and severity of a CT metal artifact, neither visually nor objectively [7]. By combining objective phantom study and visual clinical assessment more aspects affecting the visually perceived image quality is covered [17].

In this study (Table 2), the mean dose levels (CTDI_vol_) were lower for spine compared to those reported by Feldhaus et al. with 11.3 ± 3.0 mGy [12] and for sacroiliac joint compared to those reported by Selles et al. with 20.05 ± 9.99 mGy [21]. These studies were conducted with different protocols and equipment from other vendors, so any direct comparison is not possible. No similar studies were found on dose levels for the forearm. There were no correlation between higher CTDI_vol_ values and objective nor visual assessment scores (Table 2). As the highest scored protocols in the VIA had the lowest mean CTDI_vol_ for both forearm and sacroiliac joint images. The large difference in CTDI_vol_ in one forearm patient of 5.8 mGy for Siemens protocols and 10.4 mGy for Canon protocols was due to different patient positioning.

Regarding the phantom study, both iMAR and SEMAR images had lower ΔHU and ΔSD than other protocols in the phantom study. kVp settings had less impact on the MAR software-based protocols compared to non-MAR and Sn-based protocols (Fig. 4a). Especially for SEMAR, ∆ values were lowest at 80 kVp in several cases. Compared to iMAR, SEMAR seems slightly more susceptible to artifacts from stainless steel. While UHR showed the highest ∆ values of all protocols in this study, the ∆ values drops when combining 150 kVp and Sn filter, proving that the Sn filter in combination with 150 kVp provides metal artifact reduction. Adding iMAR to 150 kVp Sn scans provided little difference in the ∆ values. This might be due to fewer or no areas with artifacts for the algorithm to replace lost data.

The ∆HU when using titanium or stainless were below 20 HU for nearly all the protocols (Fig. 4a, b). In a study carried out by Hakvoort et al. [18], lower HU and SD variations were reported for titanium implants compared to stainless steel implants using a Siemens iMAR protocol with 140 kVp. This was however not the case for all protocols in this study, which might be due to different metal implant size and ROI placement. In a similar phantom study by Higashigaito et al. [19], iMAR with 120 kVp also showed the best performance for HU and SD for both titanium and stainless steel, when compared to high kVp monochromatic dual energy images.

Comparing the non-metal images from the phantom study and objective clinical results, several similarities can be seen. The higher kVp in Sn-based protocols lead to lower HU values in bone and higher HU values in soft tissue compared to SEMAR images. Adding iMAR to Sn images does not significantly alter the HU or SD values. The Sn plus UHR and SEMAR images had higher noise than Sn only images. The differences were however, smaller in the objective clinical study compared to the phantom study (Fig. 5) due to larger artifacts in the clinical study.

In the clinical study, protocol 2 received highest mean VIA score of all three readers for the criteria 2, 3, 4, and 6. The second highest median VIA score for the same criteria (Table 3) was for sacroiliac joint. The measured SD in these body parts on bone and soft tissue images were also lowest using protocol 2 compared to the other protocols used in this study. Similar results were reported in the studies carried out by Huber et al. [20] and Hackenbroch et al. [21]; 150 kVp Sn images with the lowest HU and SD measurements in bone compared to MAR and dual energy images, resulted in the highest scores in the visual image assessments.

Regrading forearm imaging, protocol 1 achieved the highest reader score with lower mean HU and SD for this body part compared to other protocols. However, SD in soft tissue was two times higher in these images than the images in protocol 4 receiving the lowest reader score. Protocol 1 also had the highest noise measured in the phantom study (Fig. 3c, d). In a study performed by Kawashima et al. [11], using an UHR filter for image reconstruction with a very sharp bone kernel, this resulted in higher noise and were similar to the results of the current study. In studies carried out by Burghardt et al. [22] and Zhang et al. [23], the UHR filter improved visualization of bone in upper and lower extremities as a result of higher spatial resolution. It was reported that the higher noise was an acceptable trade off due to improved spatial resolution. In this study, however, the improved spatial resolution did not result in higher scores in the visualization of periprosthetic cortical bone (criterion 3) compared to the other protocols. As seen in Fig. 7a, the amount of metal likely affected this score as the score improved in images with less metal.

Several studies [7, 9, 12, 13, 15] investigating metal artifact reduction report that Siemens iMAR and Canon SEMAR decreases both mean HU and SD in areas affected by metal artifacts, during the interpolation process when reconstructing images, which was also the case for the measurements in soft tissue in the present study. However, as shown by the results of the VIA, lower SD does not necessarily lead to higher visual scores. As seen in Fig. 7b, Siemens protocol 3* and protocol 6 images of sacroiliac joints were rated significantly higher in patients who had Rialto screws, compared to iFuse in the same patient group (Tables 2 and 3), due to artifacts induced by the algorithms. Selles et al. [24] reported similar artifacts with the Philips O-MAR algorithm in a clinical study with the same iFuse screws.

The main limitation in this study has been the variety of different metal implants across different body parts, resulting in few patients with identical metal compositions due to low number of patients with identical implant in our department. As a result, several different scan protocols were included. Though the basis for the different protocols (Sn *versus* MAR software) were identical, other scan parameters such as different levels of iterative reconstruction could have affected the results and make a direct comparison between the phantom study and clinical study difficult. The iMAR preset “Spine implants” was not investigated in the phantom study. It was not possible to place ROIs in the direct path of artifacts streaks for all patients in the clinical study.

In conclusion, iMAR and SEMAR were successful at replacing lost data resulting in better HU and SD values in objective measurements. The introduction of new artifacts and altering of the visual appearance of metal made some of the images unreadable and less trustworthy, resulting in lower VIA scores. All Siemens Sn protocols (protocols 1, 2, and 3) combined with 150 kVp showed similar objective MAR in the phantom study, and better objective MAR and significantly better quality at VIA than SEMAR, especially in bony structures, visualization of metal and overall diagnostic confidence in the clinical study. As the combination of Sn and UHR seems more susceptible to metal artifacts than Sn only protocols and did not score higher in criteria related to bone, we recommend using a Sn plus 150 kVp protocol as a standard on the body parts with metal implants included in this study, as it gives the possibility to reconstruct images with iMAR in case of large photon starvation artifacts.

## Data Availability

The datasets used and/or analyzed during the current study are available from the corresponding author on reasonable request.

## Abbreviations

CT: Computed tomography
CTDI_vol_: CT dose index volume
HU: Hounsfield units
iMAR: Iterative metal artifact reduction
MAR: Metal artifact reduction
PACS: Picture archiving and communication system
ROI: Region of interest
SD: Standard deviation/noise
SEMAR: Single-energy metal artifact reduction
Sn: Tin filter
UHR: Ultra-high resolution
VIA: Visual image assessment
